# New Term to Quantify the Effect of Temperature on *p*H_*min*_-Values Used in Cardinal Parameter Growth Models for *Listeria monocytogenes*

**DOI:** 10.3389/fmicb.2019.01510

**Published:** 2019-07-03

**Authors:** Veronica Martinez-Rios, Elissavet Gkogka, Paw Dalgaard

**Affiliations:** ^1^National Food Institute (DTU Food), Technical University of Denmark, Lyngby, Denmark; ^2^Arla Innovation Centre, Arla Foods Amba, Aarhus, Denmark

**Keywords:** predictive microbiology, mathematical modeling, model validation, product development, risk assessment, food safety

## Abstract

The aim of this study was to quantify the influence of temperature on *p*H_*min*_-values of *Listeria monocytogenes* as used in cardinal parameter growth models and thereby improve the prediction of growth for this pathogen in food with low pH. Experimental data for *L. monocytogenes* growth in broth at different pH-values and at different constant temperatures were generated and used to determined *p*H_*min*_-values. Additionally, *p*H_*min*_-values for *L. monocytogenes* available from literature were collected. A new *p*H_*min*_-function was developed to describe the effect of temperatures on *p*H_*min*_-values obtained experimentally and from literature data. A growth and growth boundary model was developed by substituting the constant *p*H_*min*_-value present in the Mejlholm and Dalgaard (2009) model (J. Food. Prot. 72, 2132–2143) by the new *p*H_*min*_-function. To obtain data for low pH food, challenge tests were performed with *L. monocytogenes* in commercial and laboratory-produced chemically acidified cheese including glucono-delta-lactone (GDL) and in commercial cream cheese. Furthermore, literature data for growth of *L. monocytogenes* in products with or without GDL were collected. Evaluation of the new and expanded model by comparison of observed and predicted μ_*max*_-values resulted in a bias factor of 1.01 and an accuracy factor of 1.48 for a total of 1,129 growth responses from challenge tests and literature data. Growth and no-growth responses of *L. monocytogenes* in seafood, meat, non-fermented dairy products, and fermented cream cheese were 90.3% correctly predicted with incorrect predictions being 5.3% fail-safe and 4.4% fail-dangerous. The new *p*H_*min*_-function markedly extended the range of applicability of the Mejlholm and Dalgaard (2009) model from pH 5.4 to pH 4.6 and therefore the model can now support product development, reformulation or risk assessment of food with low pH including chemically acidified cheese and cream cheese.

## Introduction

Cardinal parameter models (CPMs) contain parameters with biological or graphical interpretation (Rosso, 1995). CPMs to predict growth and growth boundary of *Listeria monocytogenes* (CPM-*Lm*) are popular, extensively validated and widely used in the assessment and risk management of processed and ready-to-eat foods These models include terms to quantitatively describe the growth inhibiting effect of different environmental factors and each term include at least one cardinal parameter related to growth limiting conditions e.g., for temperature (T_*min*_), pH (*p*H_*min*_), and water activity (a_*w*_*min*__) (Te Giffel and Zwietering, 1999; Augustin and Carlier, 2000; Augustin et al., 2005; Zuliani et al., 2007; Mejlholm and Dalgaard, 2009; Østergaard et al., 2014; Corbion, 2017). Remarkably, available CPM-*Lm* include very different *p*H_*min*_-values ranging from 4.3 to 5.0. This can be due to differences in the mathematical terms used to estimate *p*H_*min*_-values along with different acidulants and strain variability as often suggested (Augustin et al., 2005; Aryani et al., 2015). However, the experimental conditions used to estimate *p*H_*min*_-values have been little studied quantitatively, although the minimal pH-value supporting growth is known to depend on environmental conditions including temperature (Rocourt and Buchrieser, 2007).

As for other predictive food microbiology models the performance of CPM*-Lm* can be evaluated by comparison of predicted growth responses with observed growth in foods. Often, indices of model performance, including bias (B_f_) and accuracy (A_f_) factors, are used to facilitate model evaluation and to determine the range of environmental conditions where a model can be successfully validated (Ross, 1996; Augustin et al., 2005; Østergaard et al., 2014). Mejlholm et al. (2010) evaluated the performance of four of the more extensive CPM-*Lm*, including the effect of several environmental factors, by using 1,014 growth responses in meat, seafood, poultry and non-fermented dairy products. The model of Mejlholm and Dalgaard (2009) performed better than the other models, with B_f_- and A_f_- values for growth rate predictions of 1.0 and 1.5, respectively. However, the range of applicability for this model has been limited to pH-values above 5.4 as predicted growth rates at lower pH-values were too low due to a constant *p*H_*min*_-value of 4.97 used in the pH-term (Mejlholm et al., 2010; Mejlholm and Dalgaard, 2015). *L. monocytogenes* can grow at pH values as low as 4.3–4.4 (Farber et al., 1989; ICMSF, 1996; Tienungoon et al., 2000), which is important for several types of food including products acidified with glucono-delta-lactone (GDL) and gluconic acid (GAC). El-Shenawy and Marth (1990) found growth of *L. monocytogenes* in milk containing GDL or GAC at pH lower than 5.0 when products were stored at 13 and 35°C. Genigeorgis et al. (1991) showed that *L. monocytogenes* has the potential to grow in cottage cheese with pH 4.9 to 5.1 when stored at 4, 8, or 30°C. More recently, Nyhan et al. (2018) showed that béarnaise sauce and zucchini purée with pH of 4.7 can support growth of *L. monocytogenes* at 30°C. To assess and manage *L. monocytogenes* growth in food with pH as low as 4.3–4.4 it is interesting to study the performance of predictive models. Furthermore, it remains unclear if GDL or GAC have any antimicrobial effect beyond that of lowering product pH.

The objective of the present study was to quantify the influence of temperature on *p*H_*min*_-values of *L. monocytogenes* as used in CPMs. Firstly, the growth inhibiting effect of pH and GAC was studied at different temperatures to determine values for *p*H_*min*_ and the minimum inhibitory concentration (MIC) of undissociated GAC (*MI*C_*GACu*_). Secondly, a new *p*H_*min*_-function was developed, including the effect of temperature on *p*H_*min*_-values, and this new *p*H_*min*_-function was included in the growth and growth boundary model of Mejlholm and Dalgaard (2009) along with a GAC-term containing the MIC-value for undissociated GAC. Finally, the performance of the expanded model was evaluated by comparison of predicted and observed growth for *L. monocytogenes*. Data included new challenge test with chemically acidified cheese and cream cheese as well as available growth responses from literature.

## Materials and Methods

### Bacterial Strains, Pre-culture Conditions, and Inoculation

Eight strains of *L. monocytogenes* from milk, cheese, butter or the dairy environment were provided by Arla Foods and used as a cocktail (SLU 92, 612, LM 19, 6) or individually (ISO 570, 99714, SLU 2493, SLU 2265) to determined μ_*max*_-values in broth and/or for inoculation of challenge tests. Each strain was transferred from storage at −80°C to Brain Heart Infusion (BHI) broth (CM1135, Oxoid, Hampshire, UK) and incubated for 24 h at 25°C. Subsequently, for broth studies all strains were pre-cultured 1 or 2 days at 8 to 20°C in BHI broth with 0.5% NaCl and pH 5.5. For challenge tests the individual strains, later used as a cocktail, were pre-cultured one or 2 days at a temperature ranging from 8 to 20°C in BHI broth with pH 5.5 and 3% NaCl or at pH 5.2 with 1% NaCl and 500 ppm lactic acid to simulate conditions encountered in chemically acidified and cream cheese as used in the present study. Pre-cultures were grown to a relative increase in absorbance (540 nm) of 0.05 to 0.2 (Novaspec II, Pharmacia Biotech, Allerød, Denmark) equivalent to late exponential phase-beginning stationary phase. The *L. monocytogenes* cocktail of strains (*Lm-mix*) were obtained by mixing equal volumes of individual pre-cultured strains. For *Lm-mix* and pre-cultures of individual strains the cell concentration was determined by direct phase contrast microscopy prior to dilution and subsequent inoculation of experiments.

### Cardinal Parameter Values for pH and Gluconic Acid

The effect of pH and GAC concentrations on μ_*max*_-values of *L. monocytogenes* were determined at different temperatures. For each condition, growth of *Lm-mix* or individual strains was determined in duplicate by automated absorbance measurements at 540 nm (BioScreen C, Labsystems, Helsinki, Finland). Detection times defined as the incubation time necessary to observe an increase in absorbance of 0.05 from the lowest absorbance measured in the beginning of incubation; was determined for each absorbance growth curve. μ_*max*_*-*values of *Lm-mix* and individual strains were determined from absorbance detection times for serially diluted inoculation levels of 10^2^, 10^3^, 10^4^, 10^5^, and 10^6^ cfu/ml as previously described (Dalgaard and Koutsoumanis, 2001).

The effect of 17 pH-values from 4.4 to 6.8 on μ_*max*_-values were determined separately at different temperatures (5, 8, 10, 15, 20, 25, 35, and 37°C) by using BHI broth adjusted to the desired pH values with HCl, autoclaved (121°C, 15 min.) and pH readjusted if necessary. A total of 221 μ_*max*_-values, all above zero h^−1^, were determined experimentally in BHI-broth. Seventeen *p*H_*min*_-values were estimated by fitting Equation (1) to square root transformed μ_*max*_-values from broth experiments obtained for the studied pH range at different constant temperatures.

(1) $$\begin{array}{r} \sqrt{\mu_{\max}}=\;\sqrt{\mu_{ref-1\left({*}^{\circ }\text{C}\right)}\cdot \left(1-10^{\left(pH_{\min}-pH\right)}\right)} \end{array}$$

where μ_*max*_ is the maximum specific growth rate (h^−1^) and μ_*ref*−1_(^*°^C) is the fitted reference maximum specific growth rate for each studied temperature. Additionally, 44 *p*H_*min*_-values (obtained in broth adjusted to different pH-values with HCl or H_2_SO_4_) were extracted from literature and used to model the effect of storage temperature on *p*H_*min*_-values of *L. monocytogenes* (George et al., 1988; Ryser and Marth, 1988; Farber et al., 1989; Petran and Zottola, 1989; Duffy et al., 1994; Brocklehurst et al., 1995; Koutsoumanis et al., 2004a; Aryani et al., 2015).

The effect of 54 GAC (D-gluconic acid sodium salt, G9005 Sigma-Aldrich, St. Louis, USA) concentrations [0–26.7% (w/v)] on μ_*max*_-values were determined separately at different temperatures (8, 20, and 25°C) in BHI broth adjusted with HCl to pH 5.5 after addition of the organic acid and again after autoclaving (121°C, 15 min.) the broth if necessary. In total 144 μ_*max*_-values, all above 0 h^−1^, were determined experimentally in BHI-broth. Cardinal parameter value for undissociated gluconic acid (*MI*C_*GAC*_*U*__) was determined from concentrations of undissociated GAC calculated by using Equation (2) with a pK_a_-value of 3.7 (Quitmann et al., 2014). The cardinal parameter values (*MI*C_*GACU*_ and T_*min*_) were estimated by fitting Equation (3) to the 144 square root transformed μ_*max*_-values.

(2) $$\begin{array}{r} Undissociated\;organic\;acid\;\left(mM\right)=\;\frac{Organic\;acid\;\left(mM\right)}{1+\;10^{pH-pka}} \end{array}$$

(3) $$\begin{array}{r} \sqrt{\mu_{\max\;}}=\;\sqrt{\mu_{ref-3}\;\cdot {\left(\frac{T-T_{\min}}{T_{ref}-T_{\min}}\right)}^{2}\cdot {\left({1-\left(\frac{\left[GAC_{U}\right]}{MIC_{U\;GAC}}\right)}^{n1}\right)}^{n2}} \end{array}$$

where *T* is the temperature (°C), T_*min*_ is the theoretical minimum temperature that prevents growth. A constant T_*min*_ –value of −2.83°C was used and this parameter was not fitted (see Supplementary Table 2). [GAC_U_] is the concentrations (mM) of undissociated gluconic acid and *MI*C_*U*__*GAC*_ is the fitted MIC value (mM) of undissociated GAC that prevent growth of *L. monocytogenes*. In Equation (3), n1 was set to 1 or 0.5 and n2 was set to 1 or 2 (Dalgaard, 2009) in order to describe data most appropriately and this was determined from root mean square error (RMSE) values.

### Challenge Tests With Chemically Acidified Cheese and Cream Cheese

A total of 20 challenge tests were performed to generate *L. monocytogenes* growth data in GDL chemically acidified cheese (*n* = 12) and cream cheese (*n* = 8) for model evaluation (see section Evaluation of New pHmin-Function, GAC-Term and Models).

#### Chemically Acidified Cheese and Cream Cheese

Chemically acidified cheese was prepared from five different batches of ultra-filtrated milk concentrate (UF-conc.) provided by Arla Foods and containing 40% dry matter. Cheese was prepared in batches of 2,000 g of UF-conc. by adding different volumes of a glucono-delta-lactone solution (GDL 54%, Roquette®, Lestrem, France) and 36 ml of rennet solution (3.3% Hannilase® XP 200 NB, Chr. Hansen, Hørsholm, Denmark). For four batches of UF-conc. the salt concentration was adjusted by adding 3.5 or 5% NaCl (Merck, Kenilworth, US). In total, 11 laboratory-produced and one commercial chemically acidified cheese, with variation in salt, pH and added amount of GDL solution were studied in challenge tests (Table 1). Additionally, four batches of two types of cream cheese were purchased from a supermarket and were used in eight challenge tests (Table 2).

**Table 1:** Data obtained from challenge tests performed with chemically acidified cheese inoculated with *L. monocytogenes*.

				**Product characteristics (Avg**. **±** **SD)**^**a**^			**Growth parameter values (Avg**. **±** **SD)**				
**CT^**b**^**	**Batch**	***n*^**c**^**	***Storagetemp.(*^**°**^*C)***	**pH**	**Water phase salt (%)**	**Gluconic acid in water phase (ppm)**	***t*_***lag***_ (h)**	***RLT* (h)**	**Log *N*_***0***_ (Log cfu/g)**	**Log *N_***max***_*(Log cfu/g)**	**_μ*max*_ (h^−1^)**
1	1	3	14.0 ± 0.4	4.8 ± 0.2	7.24 ± 0.06	*43, 871*±*5, 715*	0.0 ± 0.0	0.0 ± 0.0	2.8 ± 0.1	5.6 ± 0.4	0.030 ± 0.01
2	1	3	14.2 ± 0.4	5.5 ± 0.1	7.58 ± 0.74	*24, 428*±*10, 675*	0.0 ± 0.0	0.0 ± 0.0	3.0 ± 0.2	7.3 ± 1.0	0.107 ± 0.03
3	1	3	14.1 ± 0.4	5.2 ± 0.1	7.44 ± 0.08	*32, 492*±*2, 835*	0.0 ± 0.0	0.0 ± 0.0	3.0 ± 0.1	7.7 ± 1.7	0.040 ± 0.00
4	1	3	14.0 ± 0.3	4.9 ± 0.0	11.70 ± 0.01	*41, 129*±*5, 146*	0.0 ± 0.0	0.0 ± 0.0	3.1 ± 0.2	3.0 ± 0.0	0.000 ± 0.00
5^d^	2	3	21.0 ± 1.4	4.6 ± 0.1	4.43 ± 0.28	*45, 162*±*10, 935*	0.0 ± 0.0	0.0 ± 0.0	3.5 ± 0.1	6.5 ± 0.2	0.055 ± 0.00
6	3	3	20.2 ± 0.2	4.7 ± 0.1	3.82 ± 0.39	*39, 944*±*2, 390*	142 ± 22	15.9 ± 4.3	1.1 ± 0.1	6.3 ± 0.1	0.126 ± 0.01
7	3	3	14.6 ± 0.2	4.8 ± 0.1	4.05 ± 0.05	*41, 841*±*2, 334*	142 ± 33	17.7 ± 10.9	1.0 ± 0.1	6.0 ± 0.4	0.080 ± 0.03
8	4	3	24.1 ± 0.0	4.7 ± 0.1	4.60 ± 0.31	*39, 638*±*1, 916*	46.9 ± 0.0	9.4 ± 0.0	2.6 ± 0.1	6.8 ± 0.0	0.139 ± 0.00
9	4	3	14.1 ± 0.1	4.8 ± 0.1	4.38 ± 0.19	*44, 622*±*4, 615*	50.9 ± 8.3	4.5 ± 2.1	2.6 ± 0.1	4.6 ± 0.1	0.059 ± 0.02
10	4	3	10.3 ± 0.1	4.8 ± 0.1	4.58 ± 0.08	*32, 329*±*10, 992*	0.0 ± 0.0	0.0 ± 0.0	2.5 ± 0.1	4.1 ± 0.3	0.013 ± 0.00
11	5	3	4.4–25.4	4.8 ± 0.0	4.48 ± 0.14	*29, 286*±*15, 672*	–^e^	–^e^	2.9 ± 0.1	5.9 ± 0.1	–^e^
12	5	3	5.2–25.3	4.8 ± 0.1	4.65 ± 0.19	*27, 742*±*3, 290*	–^e^	–^e^	2.7 ± 0.2	6.7 ± 0.1	–^e^

a *Avg., average; SD, standard deviation*.

b *Challenge test*.

c *Number of growth curves per challenge test (CT)*.

d *Commercial chemically acidified cheese*.

e *Not determined*.

**Table 2:** Storage conditions and product characteristics for challenge tests with cream cheese.

				**Product characteristics (Avg. ± SD)^**c**^**						**Growth parameter values (Avg. ± SD)^**c**^**			
**CT^**a**^**	**Batch**	***n*^**b**^**	***Storagetemp.(*^**°**^*C)***	**LAB (Log cfu/g)^**d**^**	**pH**	**Water phase salt (%)**	**Lactic acid in water phase (ppm)**	**Acetic acid in water phase (ppm)**	**Citric acid in water phase (ppm)**	***t_***lag***_* (h)**	**Log *N*_***0***_ (Log cfu/g)**	**Log *N_***max***_*(Log cfu/g)**	***_μmax_* (h^−1^)**
A	1	3	22.0 ± 0.2	3.9 ± 0.2	4.9 ± 0.2	2.07 ± 0.10	*3, 539*±376	980 ± 151	618 ± 3	0.0 ± 0.0	2.1 ± 0.0	<1.0 ± 0.0	0.000 ± 0.00^e^
B	2	3	4.5 ± 0.1	4.6 ± 0.3	5.1 ± 0.1	1.79 ± 0.04	*3, 102*±*1, 220*	*1, 188*±594	*2, 136*±1433	0.0 ± 0.0	1.9 ± 0.8	2.0 ± 0.1	0.000 ± 0.00^e^
C	2	3	10.1 ± 0.2	4.3 ± 0.2	5.1 ± 0.1	1.79 ± 0.04	*3, 102*±*1, 220*	*1, 188*±594	*2, 136*±1433	0.0 ± 0.0	2.1 ± 0.1	2.0 ± 0.1	0.000 ± 0.00^e^
D	2	3	14.6 ± 0.2	4.8 ± 0.1	5.1 ± 0.0	1.79 ± 0.04	*3, 102*±*1, 220*	*1, 188*±594	*2, 136*±1433	0.0 ± 0.0	2.1 ± 0.2	2.3 ± 0.0	0.000 ±, 0.00^e^
E	3	3	4.5 ± 0.1	4.4 ± 0.1	4.9 ± 0.1	1.93 ± 0.04	*5, 452*±*1, 941*	911 ± 443	*1, 954*±836	0.0 ± 0.0	3.0 ± 0.1	3.1 ± 0.2	0.000 ± 0.00^e^
F	3	3	10.1 ± 0.2	4.4 ± 0.1	4.8 ± 0.1	1.93 ± 0.04	*5, 452*±*1, 941*	911 ± 443	*1, 954*±836	0.0 ± 0.0	3.0 ± 0.1	3.0 ± 0.3	0.000 ± 0.00^e^
G	3	3	14.6 ± 0.2	3.2 ± 0.1	4.7 ± 0.2	1.93 ± 0.04	*5, 452*±*1, 941*	911 ± 443	*1, 954*±836	0.0 ± 0.0	3.0 ± 0.1	2.5 ± 1.2	0.000 ± 0.00^e^
H	4	3	4.7-14.6^f^	ND^g^	4.7 ± 0.0	1.84 ± 0.03	*10, 930*±*1, 815*	*1, 808*±485	*5, 121*±569	–^f^	1.4 ± 0.4	1.0 ± 0.0	–^f^

a *Challenge test*.

b *Number of growth curves per experiment*.

c *Avg., average; SD, standard deviation*.

d *LAB, lactic acid bacteria*.

e *No growth observed for the 30 days duration of experiment*.

f *Not determined due to dynamic storage temperatures*.

g *ND, not determined*.

#### Inoculation and Microbiological Analysis

Growth of *L. monocytogenes* in chemically acidified cheese and cream cheese was determined in 20 challenge tests including a total of 60 curves with growth or no-growth responses at constant and dynamic storage temperature (Tables 1, 2). Chemically acidified cheese and cream cheese were inoculated with 0.1% (v/w) of *Lm-mix* appropriately diluted in chilled saline water (0.85% NaCl) to obtain an initial concentration in the range of 1 to 3.5 log (cfu/g). Inoculation of chemically acidified cheese was performed in each batch of UF-conc. following addition of GDL solution. After the chemically acidified cheese was set it was packaged into 50 ± 1 g cheese containers and stored at 4.4–25.4°C during 10–30 days depending on the storage temperature (Table 1). Thirty three individual packages of cream cheese (150 g) were combined to form a 5,000 g sample which was then inoculated, re-packaged into 50 ± 1 g cheese containers and stored at 4.5–22.0°C during 30 days (Table 2). Storage temperature during challenge tests was regularly recorded by data loggers (TinytagPlus, Gemini Data Loggers Ltd, Chichester, UK). Six to 12 times during storage samplings were performed to quantify growth responses. At each sampling a container with 50 ± 1 g of cheese was analyzed and then discarded. Ten grams of cheese were diluted 10-fold with chilled physiological saline (PS, 0.85% NaCl and 0.10% Bacto-peptone) and subsequently homogenized for 30 s at normal speed in a Stomacher 400 (Seward Medical, London, UK). Ten-fold dilutions were performed with chilled PS. Aerobic viable counts (AVC) for chemically acidified cheese were enumerated by surface plating on standard plate count agar (CM0463, Oxoid, Hampshire, UK) and incubation at 25°C for 24 h. For cream cheese viable counts of lactic acid bacteria (LAB) were determined by double layer pour plating in nitrite actidione polymyxin (NAP) agar (pH 6.2) with incubation at 25°C for 72 h (Davidson and Cronin, 1973). Viable counts of *L. monocytogenes* were determined for both types of cheeses by surface plating on PALCAM agar base (CM0877, Oxoid, Hampshire, UK) with PALCAM selective supplement (SR0150, Oxoid, Hampshire, UK) and incubation at 37°C for 48 h.

#### Product Characteristics

pH was measured directly in the cheese with a PHC10801 puncture combination probe (Hach, Brønshøj, Denmark) at all times of sampling for microbiological analysis Other product characteristics of cheeses were determined by analysis of three packages (50 ± 1 g) for each treatment at the start of the challenge test. NaCl was quantified by automated potentiometric titration (785 DMP Titrino, Metrohm, Hesisau, Switzerland) and a_w_ was measured by a water activity meter (Aqua Lab model CX-2, Decagon devices Inc., Pullman, US). The concentration of lactic, acetic, citric, and gluconic acid was determined by HPLC using external standards for identification and quantification (Dalgaard and Jørgensen, 2000; Østergaard et al., 2014). Concentrations of undissociated organic acids in the products were calculated from Equation (2), using pKa values of 4.76, 3.13, 3.86, and 3.7 for acetic, citric, lactic, and gluconic acid, respectively, together with the pH and concentrations (mM) of organic acids in the water phase of foods. To determine water phase concentrations of organic acids, the dry matter content was determined by oven drying at 105°C for 24 ± 2 h. Due to the hydrophilic nature of the studied acetic, citric, lactic and gluconic acids more than 95% of their undissociated forms was assumed to be present in the water phase and partitioning between water and lipid phases of chesses was not quantified (Brocklehurst and Wilson, 2000; Mejlholm and Dalgaard, 2015; Wemmenhove et al., 2018).

#### Primary Growth Model

The integrated and log transformed logistic model with lag-time (four parameter model) or without lag-time (three parameter model) (Equation 4; Rosso et al., 1996) was fitted to all individual growth curves of *L. monocytogenes* obtained in challenge tests at constant temperature. Fitted parameter values for lag time (t_*lag*_, h), maximum specific growth rate (μ_*max*_, h^−1^) initial cell concentration (N_0_, cfu/g), and maximum population density (N_max_, cfu/g) were determined for each growth curve and data was reported as average ± standard deviation for each treatment (Table 1). An *F*-test was used to determine if the lag time was significant.

(4) $$\begin{array}{l} \begin{array}{lll} \log\left(N_{t}\right)=\log\;\left(N_{0}\right)\; & \; & if\;t<t_{lag}\\ \log\;\left(N_{t}\right)=log\left(\frac{N_{\max}}{1+\left(\left(\frac{N_{\max}}{N_{0}}\right)-1\right)\cdot \exp\left(-\mu_{\max}\cdot \left(t-\;t_{lag}\right)\right)}\right) & \; & if\;t\geq \;t_{lag} \end{array} \end{array}$$

where *t* is the storage time (h) and N_*t*_ is the cell concentration (cfu/g) at time *t*. Other parameters were described above.

### Growth Data of *L. monocytogenes* From Literature

A total of 170 growth responses of *L. monocytogenes* in milk, meat products and other foods at different temperatures were collected from literature. Growth of *L. monocytogenes* was described using the growth parameters t_*lag*_ (h), μ_*max*_ (h^−1^), N_0_ (log cfu/g), and N_*max*_ (log cfu/g) obtained by fitting growth data from graphs with Equation (4). Published growth rates available in tables were adjusted by multiplying them with a correction factor. The logistic model with delay was used as the reference model; therefore, the maximum specific growth rates estimated with the Baranyi model (Baranyi and Roberts, 1994) were multiplied by 0.97 (Augustin et al., 2005). For 60 of the 170 growth responses collected from literature one or more of the relevant product characteristics were not reported (Table 3). In 21 experiments the pH of milkshake and fresh pork were assumed to be 6.7 and 6.2, respectively. For 33 and 27 experiments with meat products 0.7% water phase lactic acid and 50 ppm nitrite were assumed to be present, respectively.

**Table 3:** Storage conditions and product characteristics in experiments (n= 170) used for evaluation of the model.

**Product**	**Food**	**References**	***n*^**a**^**	**No. of strains^**b**^**	**Temp. (^**°**^C)**	**Water phase salt (%)**	**a_w_^**c**^**	**pH**	**Acetic acid (%)**	**Diacetate (%)**	**Lactic acid (%)**	**GDL (%)**	**GAC (%)**	**Nitrite (ppm)**
Dairy	Milk	El-Shenawy and Marth (1990)	15	1	13	**0**^e^	0.999	3.7–6.4	–^d^	–^d^	–^d^	0–1	0–1.5	–^d^
	Milkshake	Salazar et al. (2018)	14	6	5–25	**0**	0.999	**6.7**	–^d^	–^d^	–^d^	–^d^	–^d^	–^d^
	Pudding	Lianou et al. (2018)	8	5	4–16	**0**	0.999	6.5	–^d^	–^d^	–^d^	–^d^	–^d^	–^d^
	UHT milk	Lobacz and Kowalik (2015)	15	2	3–15	**0**	0.999	6.7	–^d^	–^d^	–^d^	–^d^	–^d^	–^d^
Meat	Bologna	Barmpalia et al. (2005)	15	10	4–10	3.6	0.979	6.3	–^d^	0–0.2	**0.7**−2.6	0.12–0.25	–^d^	**50**
	Saveloy	Juncher et al. (2000)	12	5	5–10	1.9	0.989	6.1–6.4	–^d^	0–0.9	**0.7**−3.5	0–0.25	–^d^	60–150
	Fresh pork	Luo et al. (2015)	7	3	5–35	**0**	0.999	**6.2**	–^d^	–^d^	–^d^	–^d^	–^d^	^d^
	Mortadella	Daminelli et al. (2014)	6	2	8	5.3^f^	0.968	6.2	–^d^	–^d^	**0.7**	–^d^	^d^	**50**
	Bacon	Taormina and Dorsa (2010)	6	5	4–22	10.1–19.0^f^	0.620–0.910	5.1–5.6	–^d^	–^d^	–^d^	–^d^	–^d^	**50**
Purée	Zucchini	Nyhan et al. (2018)^g^	36	5^h^	30	3.4–10.6^f^	0.930–0.980	4.7–5.3	0–0.1	–^d^	–^d^	–^d^	–^d^	–^d^
Sauce	Béarnaise	Nyhan et al. (2018)^g^	36	5^h^	30	3.4–10.6^f^	0.930–0.980	4.7–5.3	0–0.1	–^d^	–^d^	–^d^	–^d^	–^d^

a *n, number of experiments/growth curves*.

b *Number of strains inoculated as a cocktail in experiments*.

c *Measured or calculated from the concentration of water phase salt*.

d *Information not reported*.

e *Bold type: assumed values. See explanation in section Evaluation of New pHmin-Function, GAC-Term, and Models*.

f *Calculated from aw using Resnik and Cherife (1988)*.

g *Some experiments contain propionic acid (1,2 mM)*.

h *One Listeria innocua strain was included in the inoculated cocktail of strains*.

*GDL, glucono-delta-lactone; GAC, gluconic acid*.

### Evaluation of New *pH_*min*_*-Function, GAC-Term and Models

The new *p*H_*min*_-function and GAC-term were evaluated by comparison of predicted and observed growth responses. We used this approached to establish if the expanded model of Mejlholm and Dalgaard (2009) including the new *p*H_*min*_-function and GAC-term (see section Expanded Model for Growth of *L. monocytogenes* in Different Foods) could predict growth of *L. monocytogenes* as determined in the present study for chemically acidified cheese and cream cheese with pH from 4.6 to 5.5 (*n* = 20; Tables 1, 2) as well as for a broad range of data from literature (*n* = 1,129; **Table 6**).

For predicted and observed μ_*max*_-values the calculated B_f_- and A_f_-values were evaluated as previously suggested with 0.95 < B_f_ < 1.11 indicating good model performance, B_f_ of 1.11–1.43 or 0.87–0.95 corresponding to acceptable model performance and B_f_ < 0.87 or > 1.43 reflecting unacceptable model performance (Ross, 1996; Ross et al., 2000; Mejlholm et al., 2010). A_f_-values above 1.5 was used to indicate an incomplete model or systematic deviation between observed and predicted μ_*max*_-values (Mejlholm and Dalgaard, 2013).

Predicted and observed growth and no-growth responses were assessed by calculating the percentage of all samples that were correctly predicted. Incorrect predictions were described as fail-safe (growth predicted when no growth was observed) or fail-dangerous (no growth predicted when growth was observed). The ψ-value was calculated for all predicted growth responses to indicate if they were close to the growth boundary of *L. monocytogenes* (ψ = 1.0) or well into the growth (ψ < 1) or no-growth (ψ > 1) regions. For chilled products with shelf-life of more than 5 weeks, product formulations resulting in a ψ-value > 2 has been recommended (Dalgaard and Mejlholm, 2019). Graphs with predicted and observed growth in challenge tests performed with chemically acidified cheese at dynamic storage temperature were used to assess these data.

### Statistical Analysis and Curve Fitting

Model parameters and standard errors were estimated by using GraphPad PRISM (version 8, GraphPad Software, San Diego, CA, USA). F-tests to determine significant lag times were performed using Microsoft Excel 2010 (Microsoft Corp., Redmond, WA, USA).

## Results

### Cardinal Parameter Values for pH and Gluconic Acid

**Figure 1:**
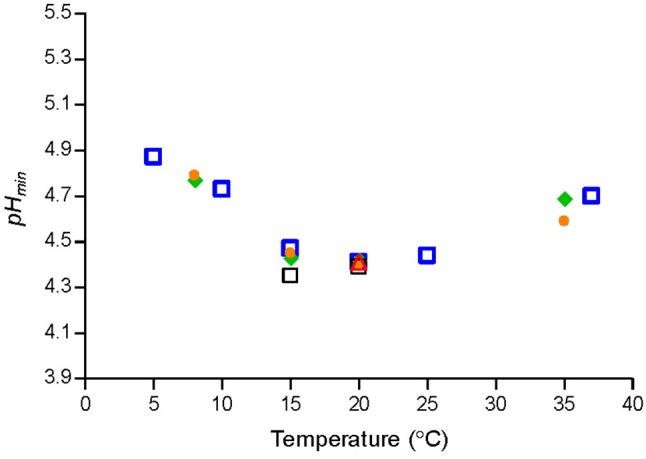
Cardinal parameter values (*p*H_*min*__)_ at different temperatures for a cocktail of *L. monocytogenes* strains [SLU 92, 612, LM19, 6, (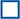)] and individual strains [ISO570 (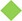), 99714 (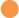), SLU 2493 (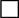), SLU 2265 (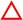)].

Temperature had a marked effect on *p*H_*min*_-values determined by fitting Equation (1) to μ_*max*_-values of *Lm-mix* or of individual strains grown in BHI broth (Figure 1). *p*H_*min*_-values on average decreased from 4.9 at 5°C to 4.3 at 15–20°C and then increased to 4.7 at 37°C (Figure 1). The cardinal parameter value for GAC i.e., the MIC-value of undissociated GAC (*MI*C_*U*__*GAC*_) was 26.4 ± 1.1 mM as determined at 8, 20, and 25°C by using Equation (3) with n1 and n2 equal to 1.

### New Cardinal Parameter *pH_*min*_*-Function for *L. monocytogenes*

**Figure 2:**
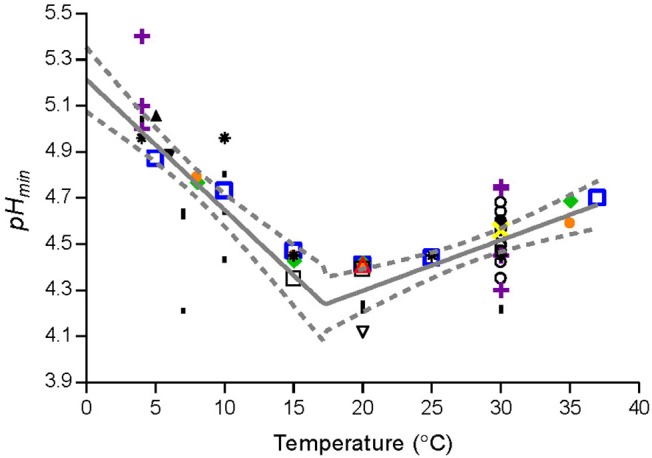
Observed and fitted *p*H_*min*_-values from the present study and from literature. Data for *L. monocytogenes* cocktail of the strains [SLU92, 612, LM19, 6, (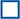)] and individual strains [ISO570 (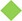), 99714 (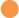), SLU 2493 (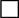), SLU 2265 (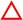)] from the present study. Data from Aryani et al. (2015) (○), Brocklehurst et al. (1995) (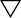), Duffy et al. (1994) (▴), Farber et al. (1989) (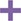), George et al. (1988) (**l**), Koutsoumanis et al. (2004a) (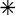), Petran and Zottola (1989) (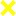), and Ryser and Marth (1988) (▾). Solid line (—) and dashed line (−−−) represent, respectively, the fitted model and confidence interval (95%).

Equation (5) was used to describe the observed effect of storage temperature on *p*H_*min*_-values of *L. monocytogenes*.

(5) $$\begin{array}{l} \begin{array}{ll} pH_{minT}=pH_{min0}-T*\left(\frac{\left(pH_{min0}-pH_{minR}\right)}{T_{R}}\right)\; & 0\leq T<T_{R\;}\\ pH_{minT}=pH_{minR}+\left(T-T_{R}\right)*\left(\frac{\left(pH_{min37}-pH_{minR}\right)}{\left(37-T_{R}\right)}\right) & T_{R}<T<37{}^{\circ}C \end{array} \end{array}$$

where T_*R*_ is the temperature (°C) corresponding to the lowest *p*H_*min*_-value; *T* is the storage temperature (°C); *p*H_*minT*_ is the estimated *p*H_*min*_–value at T (°C); *p*H_*min*0_ and *p*H_*min*37_ are, respectively, the estimated *p*H_*min*_–value at 0°C and 37°C; *p*H_*minR*_ is the *p*H_*min*_–value at T_*R*_ (°C) (Figure 2). The parameter values (Table 4) were estimated by fitting Equation (5) to *p*H_*min*_-values for *Lm*-mix, individual stains and literature data (Figure 2).

**Table 4:** Fitted parameter values for new *p*H_*min*_-function.

**Parameters**	**Values (Avg. ± SE)^**a**^**
*pH_*min*_0__*	5.2 ± 0.1
*pH_*min*_*R*__*	4.2 ± 0.0
*T_*R*_*	17.3 ± 1.3
*pH_*min*_37__*	4.7 ± 0.1

a *Avg., average; SE, standard error*.

### Expanded Model for Growth of *L. monocytogenes* in Different Foods

The model of Mejlholm and Dalgaard (2009) was expanded by substituting the constant *p*H_*min*_-value of 4.97 in the existing CPM-*Lm* by the new *p*H_*min*_-function (Equation 5) (Model 1). Model 1 was further expanded by adding a GAC-term including the *MI*C_*U*__*GAC*_–value determined in the present study (Equation 3) (Model 2). As for the model of Mejlholm and Dalgaard (2009) the effect of interaction between environmental parameters (ξ) in model 1 and model 2 was taken into account by using the Le Marc approach (Le Marc et al., 2002; Mejlholm and Dalgaard, 2009) (Supplementary Tables 1–3).

### Challenge Tests With Chemically Acidified Cheese and Cream Cheese

The chemically acidified cheese produced in the laboratory (Batch 1, 3, 4, and 5; Table 1) had pH of 4.7–5.5, water phase salt content of 3.82–11.7% and gluconic acid in the water phase of 2.44–4.46% (w/v). Commercially available chemical acidified cheese had pH 4.6 ± 0.1, water phase salt of 4.43 ± 0.28% and gluconic acid in the water phase of 4.52 ± 1.09% (Table 1). Commercial cream cheese had pH of 4.7–5.1, water phase salt content of 1.79–2.07%, lactic, acetic, and citric acid in the water phase of 3,102–10,930 ppm, 980–1,808 ppm and 618–5,121 ppm, respectively.

*L. monocytogenes* grew in the studied chemically acidified cheese with pH-values of 4.6–5.5 (Table 1). However, *L. monocytogenes* did not grow in challenge test 4 with chemically acidified cheese performed at 14.0°C due to a high water phase salt concentration (11.7 ± 0.0%) in that product. Nevertheless, growth of *L. monocytogenes* was observed in challenge test 1 with chemically acidified cheese where the product had low pH (4.8 ± 0.2) and relatively high water phase salt (7.24 ± 0.06%) (Table 1). *L. monocytogenes* did not grow in any challenge test performed with cream cheese (Table 2).

### Evaluation of Predictive Models for *L. monocytogenes*

For chemically acidified cheese and cream cheeses the original model of Mejlholm and Dalgaard (2009) predicted no-growth in 15 out of the 17 challenge tests at constant temperatures resulting in a high percentage (35%) of fail-dangerous predictions (Table 5). For the two challenge tests with pH 5.2 and 5.5 where growth was both predicted (ψ of 0.2 and 0.3) and observed the model significantly underestimated growth rates of *L. monocytogenes* as shown by a B_f_ value of 0.51 (Table 6). However, growth rates of *L. monocytogenes* in chemically acidified cheese were accurately predicted by model 1, including the new *p*H_*min*_-function (Equation 5), as shown by B_f_- and A_f_-values of 1.03 and 1.26 (*n* = 9; Table 6). Model 1 predicted growth in 9 out of the 17 challenge tests resulting in 100% correct predictions of growth and no-growth (Table 5). For challenge test with cream cheese, model 1 correctly predicted no-growth and ψ-values of 1.5 to >10 were determined showing that most of the studied products were far from the growth boundary (ψ-values of 1).

**Table 5:** Comparison of observed and predicted maximum specific growth rate (μ_*max*_-values) of *L. monocytogenes* for experimental data^a^.

**Observed**				**Predicted**		
				**Predicted growth/no-growth responses**		
	***n*^**b**^**	**Growth**	**No growth**	**Mejlholm and Dalgaard, (2009)^**c**^**	**Model 1^**d**^**	**Model 2^**e**^**
Table 1	10	9	1	2/8	**9/1**	8/2
Table 2	7	0	7	0/7	**0/7**	0/7
	Correct (%)			65	**100**	90
	Fail-safe(%)			0	**0**	0
	Fail-dangerous (%)			35	**0**	10

a *See Tables 1 and 2 for information on characteristics and storage conditions of chemically acidified and cream cheese inoculated with L. monocytogenes*.

b *n, number of experiments*.

c *Mejlholm and Dalgaard (2009) model*.

d *Model^c^ added the new pH_min_-function*.

e *Model^c^ added the new pH_min_-function and a GAC-term including MIC_GAC_U__ (mM)*.

*Bold values indicate best performing model for the evaluated data set*.

**Table 6:** Comparison of observed and predicted growth of *L. monocytogenes* obtained from experimental and literature data (*n* = 1,129).

		**Observed**		**Predicted**		
	***n*^**d**^**	**Growth**	**No growth**	**(Mejlholm and Dalgaard, 2009)^**a**^** **${B_{f}/A}_{f}^{e}$**	**Model 1^**b**^** **B_**f**_/A_**f**_**	**Model 2^**c**^** **B_f_/A_f_**
Table 1-Chemically acidified cheese	10	9	1	0.51/1.97	**1.03/1.26**	0.26/3.85
Table 2- Cream cheese	7	0	7	–^f^	**–**^f^	–^f^
Table 3-Dairy	52	48	4	0.79/10.7	**0.94/1.33**	0.92/1.31
Table 3-Meat	46	30	16	0.85/1.39	**0.91/1.39**	0.91/1.39
Meat, seafood, poultry and non-fermented dairy products ^g^	1,014	707	307	**1.00/1.49**	1.02/1.50	1.02/1.50
All data	1,129	794	335	0.98/1.50	**1.01/1.48**	1.00/1.50
		Correct (%)		89.2	90.3	89.8
		Fail-safe (%)		5.5	5.3	5.3
		Fail-dangerous (%)		5.3	4.4	4.9

a *Mejlholm and Dalgaard (2009) model*.

b *Model^a^ added the new pH_min_-function*.

c *Model^a^ added the new pH_min_-function and a GAC-term including MIC_GAC_U__ (mM)*.

d *n, number of experiments*.

e *B_f_, bias factor; A_f_, accuracy factor*.

f *B_f_/A_f_ cannot be calculated from no-growth data*.

g *Data set from Mejlholm et al. (2010)*.

*Bold values indicate best performing model for the evaluated data set*.

**Figure 3:**
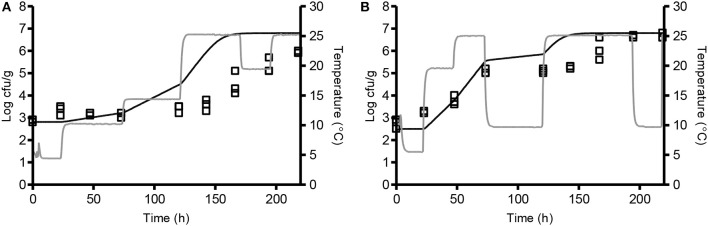
Comparison of observed (□) and predicted (-) growth of *L. monocytogenes* at dynamic storage temperature. Chemically acidified cheese was studied at 4.4–25.4°C (**A**; CT 11) and 5.2–25.3°C (**B**; CT 12). Temperature profiles are shown as gray lines. Solid lines represent the predicted growth by model 1 with N_max_ of 6.8 log cfu/g.

Model 2, developed in the present study and including the new *p*H_*min*_-function (Equation 5) as well as a GAC-term, significantly underestimated growth rates of *L. monocytogenes* in chemically acidified cheese as shown by a B_f_-value of 0.26 (*n* = 8, Table 6). The model predicted growth in 8 out of the 17 experiments resulting in 90% correct and 10% fail-dangerous predictions (Table 5). These results for evaluation of model 1 and model 2 suggest GAC, beyond lowering the pH, has no inhibiting effect on growth of *L. monocytogenes*. Inclusion of the gluconic acid MIC-term in model 2 decreased model performance and consequently this term is not needed to correctly predict growth of *L. monocytogenes* in the studied chemically acidified cheese. Further evaluation of model 1 and model 2 was performed with μ_*max*_-data obtained from the literature. The Mejlholm and Dalgaard (2009) model slightly underestimated growth rates of *L. monocytogenes* in dairy and meat products as shown by B_f_-values of 0.79 and 0.85, respectively (Table 6). For these products without GAC, similar B_f_-values were obtained with model 1 and 2 indicating that growth can be accurately predicted with both models (Table 6). However, exclusively model 1 was able to accurately predict growth in chemically acidified cheese with low pH as shown above (Table 5). Importantly, model 1 predicted growth of *L. monocytogenes* in meat, seafood, poultry and non-fermented dairy products (*n* = 707) with good precision and resulted in B_f_-/A_f_-values of 1.02/1.50 (Table 6). Model 1 and the Mejlholm and Dalgaard (2009) model were further evaluated with a data set composed by experimental and literature data (*n* = 1,129, Table 6). B_f−_ and A_f_-values for model 1 were of 1.01 and 1.48, whereas values of 0.98 and 1.50 were obtained with the Mejlholm and Dalgaard (2009) model. Model 1 predicted growth/no-growth responses correctly for 90.3% of the growth responses with the incorrect predictions distributed as 5.3% fail-safe and 4.4% fail-dangerous, resulting in a better performance compared with either of the other two models (Table 6). Model predictions were fail-safe or correct for the two challenge tests with chemically acidified cheese stored at dynamic temperature. An N_max_-value of 6.8 log cfu/g was used for these predictions as this value was observed in products with similar characteristics (Table 1, Figure 3). For zucchini purée and béarnaise sauce, with low pH and storage at 30°C, model 1 had an acceptable B_f−_value of 1.26 but the A_f_-values of 1.56 and 38% fail-safe prediction indicated unacceptable precision of the model (Table 7).

**Table 7:** Observed and predicted growth of *L. monocytogenes* with data from Nyhan et al. (2018).

		**Observed growth**		**Predicted growth (Model 1^**a**^)**	**Predicted growth (Model 1**^**a**^ **with propionic acid**^**b**^**)**			
	***n*^**c**^**	**Growth**	**No growth**	**${B_{f}/A}_{f}^{d}$**	**${B_{f}/A}_{f}^{d}$**	**Correct (%)**	**Fail-safe (%)**	**Fail-dangerous (%)**
Total (Zucchini purée and Béarnaise sauce)	72	36	36	1.26/1.56	**1.14/1.49**	63	38	0

a *Mejlholm and Dalgaard (2009) model including new pH_min_-function*.

b *Propionic acid MIC value from Le Marc et al. (2002)*.

c *n, number of experiments*.

d *B_f_, bias factor; A_f_, accuracy factor*.

*Bold value indicates best performing model for the evaluated data set*.

## Discussion

The present study quantified the effect of temperature on *p*H_*min*_-values for *L. monocytogenes* and included this effect (Equation 5) in an extensive growth and growth boundary model that was subsequently successfully validated for pH values as low as 4.6 (Supplementary Tables 1, 2). This expanded model (Model 1, section Expanded Model for Growth of *L. monocytogenes* in Different Foods) including the effect of both general product characteristics (temperature, NaCl/aw, pH) and product specific ingredients (organic acids and other preserving factors) provides new options to predict *L. monocytogenes* growth responses. These predictions are useful in the assessment and management of *L. monocytogenes* growth for processed and ready-to-eat foods including non-fermented dairy products and cream cheese with pH of 4.6 or above. Based on the performed model evaluation, the range of applicability for model 1 in foods includes storage temperatures from 2 to 35°C, pH between 4.6 and 7.7 and water phase salt concentrations as low as 0% with the range of the other environmental factors as reported previously (Mejlholm et al., 2010; Mejlholm and Dalgaard, 2015).

The successfully validated model 1 can be used to assess *L. monocytogenes* growth in chemically acidified cheeses and cream cheeses depending on storage conditions and product characteristics. As an example, if a chemically acidified cheese (pH 4.6 and 4.4% water phase NaCl) is contaminated with 1 *L. monocytogenes*/g after pasteurization (e.g., while adding GDL) and subsequently chill stored at 5°C then the product will not support growth. However, if the product is stored at 25°C a critical concentration of 100 cfu/g (CA, 2011; EC, 2011; ANZ, 2018) will be exceeded after <2 days. Model 1 predicts that a formulation with 0.21% lactic acid in the water phase will prevent growth of *L. monocytogenes* for that product also at 25°C (ψ of 2.5). As another example the model can be used to predict growth/no-growth-conditions for cream cheese at 5°C with pH 5.2, 1.9% water phase NaCl, water phase organic acids concentrations of 0.20% (lactic), 0.10% (acetic), and 0.10% (citric). If the product is contaminated with 1 cfu/g then growth of *L. monocytogenes* will not be supported (ψ of 2.1); however if the same contaminated product is stored at 25°C then the critical cell concentration will be exceeded in 2.5 days (ψ of 0.4). Model 1 predicted that a cream cheese reformulated with lower pH (5.0) and increased concentrations in the water phase of lactic acid (0.45%) and acetic acid (0.15%) will prevent growth of *L. monocytogenes* at 25°C (ψ of 2.4).

The observed effect of temperature on *p*H_*min*_-values for *L. monocytogenes* (Figure 1) are in agreement with previous studies based on broth acidified with hydrochloric acid. Koutsoumanis et al. (2004a) found that the minimum pH supporting growth of *L. monocytogenes* at 4 and 10°C was 4.96, while at 15 and 30°C it was 4.45. Farber et al. (1989) determined pH of 5.0 to 5.4 needed to prevent *L. monocytogenes* growth at 4°C whereas at 30°C lower pH-values of 4.3 to 4.7 were required. For a_w_ of 0.990, 0% lactic acid and temperatures of 4, 15, and 30°C the model of Tienungoon et al. (2000) predicted pH-growth-limits of *L. monocytogenes* to be 5.38, 4.40, and 4.38. These data are in agreement with the present study, where the effect of temperature on *p*H_*min*_-values was quantified with markedly more data. Furthermore, the new model 1 includes more environmental factors than the model of Tienungoon et al. (2000) and therefore has wider application e.g., for product formulation or documentation of food safety.

The effect of temperature on *p*H_*min*_-values for *L. monocytogenes* as quantified in the present study (Figure 1) has been important to accurately predict growth and growth boundary of this pathogen in food with low pH (Tables 5, 6). Temperature may have a similar effect on other microorganisms than *L. monocytogenes* as indicated by growth data for e.g., *Escherichia coli* (Salter et al., 2000; McKellar and Lu, 2001), *Salmonella* (Koutsoumanis et al., 2004b) and *Staphylococcus aureus* (Valero et al., 2009). It seems interesting in future studies to evaluate if CPMs with temperature dependent *p*H_*min*_-function could be valuable to predict growth and growth boundary responses of other microorganisms as well as to obtain more information on why a minimum *p*H_*min*_-value is observed at a temperature markedly below the optimum temperature for growth for *L. monocytogenes*.

The performed experiments with chemically acidified cheese highlighted an important limitation of the Mejlholm and Dalgaard (2009) model to accurately predict growth of *L. monocytogenes* in foods with low pH (Tables 5, 6). This limitation is due to a constant *p*H_*min*_-value for *L. monocytogenes* of 4.97 and consequently, no-growth is predicted below that pH-value, irrespective of the storage temperature. Model 1, with a new *p*H_*min*_-function (Equation 5), did not have this limitation and showed good model performance for products with pH as low 4.6 (Table 6).

The acceptable B_f_-value but high A_f_-value of model 1 for zucchini purée and béarnaise sauce (B_f_- and A_f_-values of 1.26 and 1.56; Table 7) could be due to inhibiting compounds in some of these products that were not included in model 1. In fact, some of the treatments studied by Nyhan et al. (2018) included propionic acid. It was therefore investigated if including a propionic acid term and MIC value from Le Marc et al. (2002) could improve the performance of model 1. Addition of the Le Marc et al. (2002) propionic acid term and MIC value improved performance of the expanded model 1 (B_f_- and A_f_-values of 1.14 and 1.49; Table 7), however, further evaluation of the expanded model containing a propionic acid term is necessary for vegetable products and sauces due to a high percentage of fail-safe predictions (38%; Table 7).

Despite the inhibitory effect of GAC observed in broth, with *MI*C_*U*__*GAC*_ of 26.4 ± 1.1 mM (Section Cardinal Parameter Values for pH and Gluconic Acid), comparison of predicted and observed *L. monocytogenes* growth in foods (Table 6) showed no need to include a GAC-term in the developed growth and growth boundary model (Model 1, section Expanded Model for Growth of *L. monocytogenes* in Different Foods). This result is not in contradiction with available data although an antimicrobial effect of GDL and GAC against *L. monocytogenes* has been reported by several studies. For instance, Juncher et al. (2000) found a recipe for saveloys with 2.0% lactate and 0.25% GDL to prevent growth of *L. monocytogenes*. The addition of GDL reduced product pH from 6.37 to 6.08 resulting in an increase of undissociated lactic acid from 1.2 to 2.3 mM. Similarly, Qvist et al. (1994) found bologna-type sausage with 2% lactate and 0.5% GDL prevented growth of *L. monocytogenes* at 5 and 10°C during 28 days of storage. Product pH was reduced from 6.6 to 6.0 by 0.5% GDL and this resulted in an increase of undissociated lactic acid from 0.7 to 2.8 mM. El-Shenawy and Marth (1990) suggested that using GAC or GDL at concentrations high enough to coagulate milk for cottage cheese production should contribute to control *L. monocytogenes* during the manufacturing process. For these examples, the *L. monocytogenes* growth inhibition can be explained by the combined effect of product pH, undissociated lactic acid and other product characteristics rather than by the suggested effect of GAC or GDL as shown in the present study for different foods by using model 1.

In conclusion, the present study quantified and modeled the effect of temperature used to estimate *p*H_*min*_-values of *L. monocytogenes* and showed the importance of this effect for accurate prediction of growth in low pH foods. The new model can support product development, reformulation or risk assessment of a wide range of foods including meat, seafood and different dairy products (milk, cream, desserts, chemically acidified cheese, and cream cheese). The new model can be included in predictive microbiology application software such as the Food Spoilage and Safety Predictor (FSSP http://fssp.food.dtu.dk/) to facilitate its use by the industry and food safety authorities.

## Data Availability

All datasets generated for this study are included in the manuscript.

## Author Contributions

The literature review was conducted by VM-R and EG. Research objectives were defined by VM-R, and the specific research questions was developed by VM-R, EG, and PD. Data was analyzed by VM-R, and results were interpreted by VM-R and PD. Expansion of the model was by VM-R. The tables, figures, and manuscript were created by VM-R. All authors revised and approved the manuscript.

### Conflict of Interest Statement

EG is employed by Arla Foods. The remaining authors declare that the research was conducted in the absence of any commercial or financial relationships that could be construed as a potential conflict of interest.
