# Supplementary material for: New Term to Quantify the Effect of Temperature on pHmin-Values Used in Cardinal Parameter Growth Models for Listeria monocytogenes
Source: Front Microbiol. 2019 Jul 3;10:1510. doi: 10.3389/fmicb.2019.01510 (PMC6628878; doi:10.3389/fmicb.2019.01510)
Supplement: Supplementary file 1 [file Table_1.pdf]

Supplementary Table 1. Cardinal parameter models for *L. monocytogenes* (Eq. S1, S3 and S4) and new pH_{min}-function (Eq. S2)

References	Model	Equation
Mejlholm and Dalgaard (2009)	$\mu_{max} = \mu_{ref} \cdot \left[\frac{(T-T_{min})}{(T_{ref}-T_{min})}\right]^2 \cdot \frac{(a_w - a_{w\,min})}{(1-a_{w\,min})} \cdot \left[1-10^{(pH_{min}-pH)}\right] \cdot \left(\frac{P_{max}-P}{P_{max}}\right) \cdot \left(\frac{CO_{2\,max}-CO_{2\,equilibrium}}{CO_{2\,max}}\right) \cdot \left(\frac{MIC_{NIT}-NIT}{MIC_{NIT}}\right)^2 \cdot \left(1-\left(\frac{[AAC_U]}{[MIC_{U\,AAC}]}\right)^{n1}\right)^{n2} \cdot \left(1-\left(\frac{[CAC_U]}{[MIC_{U\,CAC}]}\right)^{n1}\right)^{n2} \cdot \left(1-\left(\frac{[DAC_U]}{[MIC_{U\,DAC}]}\right)^{n1}\right)^{n2} \cdot \left(1-\left(\frac{[LAC_U]}{[MIC_{U\,LAC}]}\right)^{n1}\right)^{n2} \cdot \xi$	S1
The present study eq. (5)	$pH_{minT} = pH_{min0} - T * \left(\frac{(pH_{min0}-pH_{minR})}{T_R}\right)$ $\quad 0 \le T < T_R$ $pH_{minT} = pH_{minR} + (T-T_R) * \left(\frac{(pH_{min37}-pH_{minR})}{(37-T_R)}\right)$ $\quad T_R < T < 37℃$	S2
The present study Model 1	$\mu_{max} = \mu_{ref} \cdot \left[\frac{(T-T_{min})}{(T_{ref}-T_{min})}\right]^2 \cdot \frac{(a_w - a_{w\,min})}{(1-a_{w\,min})} \cdot \left[1-10^{(Eq.(S2)-pH)}\right] \cdot \left(\frac{P_{max}-P}{P_{max}}\right) \cdot \left(\frac{CO_{2\,max}-CO_{2\,equilibrium}}{CO_{2\,max}}\right) \cdot \left(\frac{MIC_{NIT}-NIT}{MIC_{NIT}}\right)^2 \cdot \left(1-\left(\frac{[AAC_U]}{[MIC_{U\,AAC}]}\right)^{n1}\right)^{n2} \cdot \left(1-\left(\frac{[CAC_U]}{[MIC_{U\,CAC}]}\right)^{n1}\right)^{n2} \cdot \left(1-\left(\frac{[DAC_U]}{[MIC_{U\,DAC}]}\right)^{n1}\right)^{n2} \cdot \left(1-\left(\frac{[LAC_U]}{[MIC_{U\,LAC}]}\right)^{n1}\right)^{n2} \cdot \xi$	S3
The present study Model 2	$\mu_{max} = \mu_{ref} \cdot \left[\frac{(T-T_{min})}{(T_{ref}-T_{min})}\right]^2 \cdot \frac{(a_w - a_{w\,min})}{(1-a_{w\,min})} \cdot \left[1-10^{(Eq.(S2)-pH)}\right] \cdot \left(\frac{P_{max}-P}{P_{max}}\right) \cdot \left(\frac{CO_{2\,max}-CO_{2\,equilibrium}}{CO_{2\,max}}\right) \cdot \left(\frac{MIC_{NIT}-NIT}{MIC_{NIT}}\right)^2 \cdot \left(1-\left(\frac{[AAC_U]}{[MIC_{U\,AAC}]}\right)^{n1}\right)^{n2} \cdot \left(1-\left(\frac{[CAC_U]}{[MIC_{U\,CAC}]}\right)^{n1}\right)^{n2} \cdot \left(1-\left(\frac{[DAC_U]}{[MIC_{U\,DAC}]}\right)^{n1}\right)^{n2} \cdot \left(1-\left(\frac{[LAC_U]}{[MIC_{U\,LAC}]}\right)^{n1}\right)^{n2} \cdot \left(1-\left(\frac{[GAC_U]}{MIC_{U\,GAC}}\right)^{n1}\right)^{n2} \cdot \xi$	S4
