# Supplementary material for: New Term to Quantify the Effect of Temperature on pHmin-Values Used in Cardinal Parameter Growth Models for Listeria monocytogenes
Source: Front Microbiol. 2019 Jul 3;10:1510. doi: 10.3389/fmicb.2019.01510 (PMC6628878; doi:10.3389/fmicb.2019.01510)
Supplement: Supplementary file 2 [file Table_2.pdf]

Supplementary Table 2. Cardinal parameter values for *L. monocytogenes*.

Model parameters	Value	n1	n2
μ_{ref} (h^{-1})	0.419	-	-
T_{min} (°C)	-2.83	-	-
$a_{w\ min}$	0.923	-	-
pH_{min}	4.97	-	-
Phenol (P_{max}, ppm)	32	-	-
$CO_{2\ max}$ (ppm)	3140	-	-
Nitrite (MIC_{NIT}, ppm)	350	-	-
Minimum inhibitory concentration (MIC) of undissociated organic acids (mM)			
Acetic acid (AAC)	10.3	0.5	1
Citric acid (CAC)	2.12	1	1
Diacetate (DAC)	4.80	0.5	1
Lactic acid (LAC)	3.79	1	1
Gluconic acid (GAC)	26.4	1	1
pH_{min_0}	5.2	-	-
pH_{min_R}	4.2	-	-
T_R	17.3	-	-
$pH_{min_{37}}$	4.7	-	-
