# Supplementary material for: New Term to Quantify the Effect of Temperature on pHmin-Values Used in Cardinal Parameter Growth Models for Listeria monocytogenes
Source: Front Microbiol. 2019 Jul 3;10:1510. doi: 10.3389/fmicb.2019.01510 (PMC6628878; doi:10.3389/fmicb.2019.01510)
Supplement: Supplementary file 3 [file Table_3.pdf]

Supplementary Table 3. Effect of interaction between environmental factors (ξ) in Model 1[a].

$\xi\left(\varphi(T, a_w, pH, P, CO_2, NIT, [AAC], [CAC], [DAC], [LAC])\right)$ $\xi = \begin{cases} 1 & ; \quad \psi \leq 0.5 \\ 2(1-\psi) & ; \quad 0.5 \leq \psi \leq 1 \\ 0 & ; \quad \psi \geq 1 \end{cases}$	S5
$\varphi_T = \left[1 - \left(\frac{T - T_{min}}{T_{ref} - T_{min}}\right)\right]^2$ $\varphi_{a_w} = \left[1 - \sqrt{\frac{a_w - a_{w\,min}}{1 - a_{w\,min}}}\right]^2$ $\varphi_{pH} = \left[1 - \sqrt{(1 - 10^{(Eq.(S2) - pH)}}\right]^2$ $\varphi_P = \left[1 - \sqrt{\frac{(P_{max} - P)}{P_{max}}}\right]^2$ $\varphi_{CO_2} = \left[1 - \sqrt{\frac{(CO_{2\,max} - CO_{2\,equilibrium})}{CO_{2\,max}}}\right]^2$ $\varphi_{NIT} = \left[1 - \left(\frac{MIC_{NIT} - NIT}{MIC_{NIT}}\right)\right]^2$	S6
$\varphi_{[AAC]:[CAC]:[DAC]:[LAC]} = \left\{1 - \left[\left(1 - \sqrt{\frac{[AAC_U]}{MIC_{U\,AAC}}}\right) \cdot \left(1 - \frac{[CAC_U]}{MIC_{U\,CAC}}\right) \cdot \left(1 - \sqrt{\frac{[DAC_U]}{MIC_{U\,DAC}}}\right) \cdot \left(1 - \sqrt{\frac{[LAC_U]}{MIC_{U\,LAC}}}\right)\right]\right\}^2$	S7
$\psi = \sum_i \frac{\phi_{e_i}}{2\prod_{j \neq i}\left(1 - \phi_{e_j}\right)}$	S8

[a] The effect of interaction between environmental factors (ξ) was modelled as described by Le March et al. (2002). The value of ξ was calculated according to equation S5, with contributions from terms for the different environmental factors as shown in equation S6 and S7. In equation S8, e_i represents the environmental factors. Equation S5 divides the space of environmental factors into three regions: (i) if ψ is less than 0.5 then no interactive effect between the effect of environmental factors occurs (ξ = 1); (ii) if ψ is greater than 1 there is no growth (ξ = 0); and (iii) if ψ is less than 1 and greater than 0.5 then the growth rate (μ_{max}, h^{-1}) is reduced depending on the value of ψ (Le March et al., 2002; Mejlholm and Dalgaard, 2009).
